# Eryptosis as a New Insight in Malaria Pathogenesis

**DOI:** 10.3389/fimmu.2022.855795

**Published:** 2022-05-13

**Authors:** Aline Miranda Scovino, Paulo Renato Rivas Totino, Alexandre Morrot

**Affiliations:** ^1^ Instituto de Microbiologia Paulo de Góes, Universidade Federal do Rio de Janeiro, Rio de Janeiro, Brazil; ^2^ Laboratório de Pesquisa em Malária, Instituto Oswaldo Cruz (FIOCRUZ), Rio de Janeiro, Brazil; ^3^ Laboratório de Imunoparasitologia, Instituto Oswaldo Cruz (FIOCRUZ), Rio de Janeiro, Brazil; ^4^ Faculdade de Medicina, Universidade Federal do Rio de Janeiro, Rio de Janeiro, Brazil

**Keywords:** eryptosis, *Plasmodium* infection, malaria pathogenesis, immunopathology, severe malarial anemia

## Abstract

Eryptosis is a programmed cell death-like process that occurs in red blood cells. Although the red blood cells are anucleated, there are similarities between eryptosis and apoptosis, such as increased calcium efflux, calpain activation, phosphatidylserine exposure, cell blebbing and cell shrinkage. Eryptosis occurs physiologically in red blood cells, as a consequence of the natural senescence process of these cells, but it can also be stimulated in pathological situations such as metabolic syndromes, uremic syndromes, polycythemia vera, anemias such as sickle cell anemia and thalassemia, and infectious processes including *Plasmodium* infection. Infection-induced eryptosis is believed to contribute to damage caused by *Plasmodium*, but it’s still a topic of debate in the literature. In this review, we provided an overview of eryptosis mechanisms and its possible pathogenic role in malaria.

## Introduction

Apoptosis is a program cell death process that is a common mechanism among many cell types in physiology and disease. Apoptosis typically does not induce inflammation, or tissue scaring (1). The intrinsic pathways and machinery involved in this process is already well understood. A series of genetically regulated steps are essential for the process, with the activation of pro-apoptotic genes, biochemical and morphological changes within cells. These includes DNA fragmentation, cell surface externalization of phosphatidylserine and activation of caspases. In mammalian cells there are two major pathways to caspase-mediated cell death: the extrinsic and the mitochondria-dependent pathway (2).

Despite the absence of nucleus and mitochondria, in 2001, Bratosin and collaborators described a process similar to apoptosis occurring in red blood cells, later called eryptosis. Eryptosis has several similarities with apoptosis, regardless of the trigger, induction of an eryptotic state generally involves the entry of extracellular calcium into the cell, with activation of caspases and calpains, which induce membrane phosphatidylserine exposure, cell blebbing and cell shrinkage (3).

Eryptosis has been associated with several pathologies and can be triggered by several signals, including extended storage after blood donation (4), osmotic shock (5), xenobiotics, oxidative stress and energy depletion (6). Osmotic shock as well as oxidative stress induce the formation of Prostaglandin E_2_ (PGE_2_) that activates a calcium-permeable cation channel in the erythrocyte membrane, increasing the cytosolic calcium content, while diminishing chloride content and, consequently, induces cell shrinkage and phosphatidylserine exposure (7). During hyperosmotic shock, p38 MAPK (Mitogen-activated protein kinase) is expressed in erythrocytes and participates in the machinery triggering eryptosis (8).

One of the first events during eryptosis is the influx of calcium into the erythrocyte. In physiological situations, its concentration is much lower than the concentration of calcium in the blood plasma, at least 40,000 times (9). After stimulation, such as hyperosmotic shock and oxidative stress, the calcium channels present in the erythrocyte membrane open, increasing their influx into the cell (9).

These differences of calcium concentrations are maintained by the enzyme Plasma Membrane Ca^2+^ Pump (PMCP), with energy expenditure. This enzyme is able to sense the increase in cytosolic calcium levels through the calcium/calmodulin complex. Upon activation, calcium influx occurs mainly through nonspecific ion channels, as the transient receptor potential channel TRPC6, which is expressed in erythrocytes (10). Once into the cell, calcium binds to several proteins, associated with different cellular, physiological or pathophysiological processes, including clamodulin, which forms a complex with calcium regulating the function of several other proteins, such as the cytoskeletal proteins, PMCP itself, endothelial NO synthase (eNOS) and protein kinase C (PKC) (11) that will participate in the mechanisms of eryptosis (12).

The externalization of phosphatidylserine is also an event dependent on calcium concentrations. The proteins responsible for the symmetry of phosphatidylserine in the cellular membrane are calcium-dependent proteins. Flipases are examples of transmembrane proteins responsible for translocating phospholipids, maintaining plasma membrane asymmetry. They translocate phosphatidylserine from the outer to the inner side of the membrane. The scramblases are a distinct form of flipases, but also translocate phospholipids between the two monolayers of a lipid bilayer of a cell membrane. Scramblases translocate phosphatidylserine in the opposite direction to the translocation made by flipases, from the inside out. Therefore, exposure of phosphatidylserine in the outer layer of the plasma membrane is a consequence of high levels of calcium that inhibit flipases activity and activate scramblases (9).

Another important events during eryptosis is cell shrinkage and blebbing, which is also controlled by calcium influx. Increased cytosolic calcium activity further activates the cysteine endopeptidase calpain, an enzyme degrading the cytoskeleton and thus leading to cell membrane blebbing (13). The high calcium concentration induces the formation of the calcium/calmodulin complex that is also associated with potassium channels called Gardos channels. This channel induces the hyperpolarization of the membrane which in turn promotes the efflux of chlorine, potassium and water, resulting in cell shrinkage (9).

Ceramide is a sphingolipid that stimulates eryptosis. It is generated after osmotic shock by breaking sphingomyelin and works as a sensitizer to the effect of calcium influx. Ceramide is located in lipid rafts and destabilizes the interaction of cytoskeleton proteins with the plasma membrane, increasing membrane instability, and facilitating the erythrocyte cell membrane scrambling and exposure of phosphatidylserine (13).

Caspase activation is another event that occurs during eryptosis, however unlike what occurs in nucleated cells, in erythrocytes, caspase activation is not a central event. Calcium influx and calcium-induced phosphatidylserine exposure do not appear to be dependent on caspase activation (6). Although there are studies showing caspase 3 activation associated with phosphatidylserine exposure in erythrocytes under oxidative stress (14), they do not play a central role.

## Eryptosis and Physiology

Eryptosis is an event that occurs physiologically for the control and elimination of senescent red blood cells. The senescence process occurs through the accumulation of cellular damage caused by oxidative stress and the impossibility of recovering the already existing molecules. This process promotes morphological alterations in cell volume, density, and shape, as well as quantitative and qualitative changes on cell plasma membrane, making the cells targeted by the phagocytic cells in the liver and spleen, and thus being removed from the bloodstream.

Alterations reach critical levels after approximately 120 days after red blood cell entry into the circulation (15). The mechanical filtration of red blood also occurs in the spleen, due to its three-dimensional structure. In the red pulp there is a structure, smaller than capillaries, called interendothelial slit, which do not allow the passage of red blood cells whose volume, shape and density ratio are abnormal. In this way, red blood cells whose geometric patterns are modified, such as during eryptosis, are retained in the organ (16).

Red blood cells also present surface molecules that signal their state of senescence, indicating the right moment when they should be eliminated. Oxidative stress, for example, promotes the formation of band 3 protein aggregates (one of the most abundant transmembrane proteins in red blood cells), which, when stabilized by oxidized hemoglobin molecules (hemicromes), are recognized as antigens by autologous IgG antibodies and complement system. With the deposition of a critical density of antibodies and complement molecules, senescent red blood cells are recognized and eliminated by macrophages. This process also destabilizes the interaction of the cytoskeleton with the band 3 proteins, promoting the formation of vesicles, less fluidity and phospholipid scrambling of the plasma membrane (15).

Another way of eliminating senescent red blood cells is the exposure of phosphatidylserine in the outer portion of its plasma membrane, a sign that indicates that the cell must be phagocytosed, because in healthy cells this phospholipid is actively maintained in the cytoplasmic portion of the plasma membrane. Concomitantly, there is negative regulation of the CD47 molecule, a transmembrane protein whose normal expression indicates a sign of “do not eat me”. Thus, the reduction of CD47 expression and the increase of phosphatidylserine in the outer portion of the plasma membrane stimulates phagocytosis and the elimination of these red cells (17).

When erythrocytes suffer some stress or damage, before their natural senescence period, eryptosis is a possible mechanism for eliminating these damaged cells, instead of hemolysis. The lysis of red blood cells promotes the accumulation of hemoglobin and heme in the blood, which consequently react rapidly with nitric oxide (NO) reducing their availability. This reduction in NO levels can cause vasoconstriction, increased expression of adhesion molecules and endothelial activation. These adhesion molecules act as pro-inflammatory ligands of innate immune receptors, promoting platelet aggregation, and then recruiting cells of the innate response, which will initiate an inflammatory process with the release of pro-inflammatory cytokines and chemokines (18). In addition, the accumulation of hemoglobin to be filtered in the renal glomeruli can cause occlusion of them (19). Therefore, the elimination of red blood cells by eryptosis can prevent even greater damage to the organism.

## Eryptosis in Malaria

Eryptosis has also been associated with several pathologies, including metabolic syndromes, uremic syndromes, polycythemia vera (20) and anemias such as sickle cell anemia and thalassemia. Infectious diseases also induce eryptosis, including *Plasmodium* infection (21). In humans, infection by the protozoan of the genus *Plasmodium* causes malaria. Its transmission occurs through an arthropod vector, the mosquito of the genus *Anopheles*. The species that parasitize humans are: *Plasmodium falciparum*, *P. vivax*, *P. malariae*, *P. ovale* and *P. knowlesi*. However, the species responsible for most cases are *P. falciparum* and *P. vivax*. There are also four species of rodent parasitic plasmodia, which are great tools for the studies of malaria in animal models, such as testing new drugs, studies on drug resistance mechanisms, vaccine development, among others; they are: *P. yoelii*, *P. berghei*, *P. chabaudi* and *P. vinckei* (22).

The *Plasmodium* life cycle corresponds to a sexual phase in the invertebrate host, and an asexual phase in the vertebrate human host. The life cycle of *Plasmodium* in the human host begins when the female mosquito transfers the form of the parasite called sporozoite directly to the skin. After reaching the blood vessels, the sporozoites travel to the liver where they will infect the hepatocytes. In these cells, sporozoites multiply by schizogony, differentiating into schizonts full of merozoites, the infective forms of red blood cells, which will enter the bloodstream, initiating the blood stage of infection, in which signs and symptoms of the disease occur (23).

In the blood phase, the parasites multiply intraerythrocytically by schizogony, passing through three successive and morphologically distinct blood stages: the young (ring-shaped) trophozoite, the mature trophozoite and, finally, the schizont. Some blood merozoites, however, differentiate into gametocytes responsible for the continuity of the cycle in the *Anophele*s mosquito, after the blood meal in the vertebrate host (24). Clinical symptoms of malaria are associated with the blood cycle of the disease, characterized by infection and lysis of red blood cells. The disease manifests itself in an asymptomatic to mild form, with symptoms such as fever, headache, nausea, drowsiness, among others. As the infection progresses, without timely diagnosis and adequate treatment, malaria can progress to its severe forms, which mainly include severe anemia, cerebral malaria and metabolic acidosis (25).

**Figure 1:**
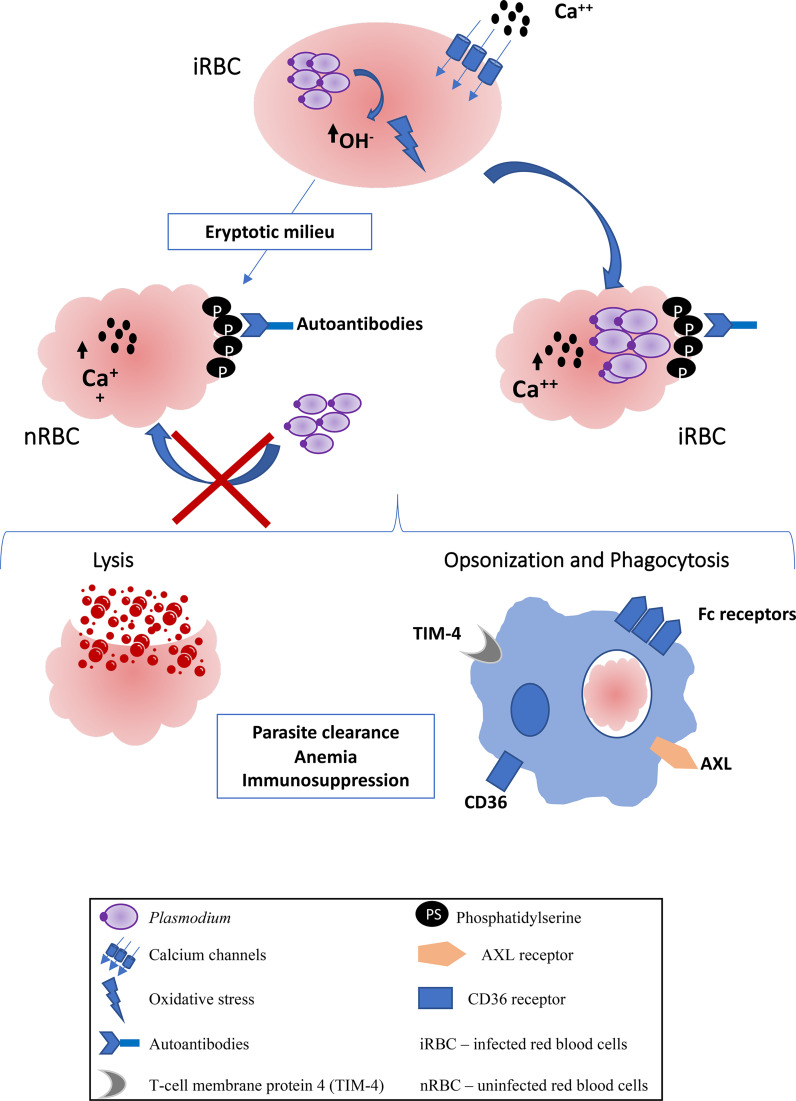
Scheme of eryptosis in *Plasmodium* infection. The metabolism of the parasite induces the production of OH-radicals that promote oxidative stress in red blood cells. There is the opening of calcium channels, an increase in intracellular calcium and, consequently, the exposure of phosphatidylserine. Uninfected red blood cells also suffer eryptosis, probably from eryptotic factors released by infected red blood cells, becoming refractory to infection. Both uninfected and infected RBCs are eliminated by lysis mediated by activation of the complement system, or by prophagocytosis mediated by Fc receptors on phagocytes, due to the presence of autoantibodies against phosphatidylserine. There are also specific receptors against phosphatidylserine that can promote uptake by phagocytes, such as CD36, TIM-4 and AXL. The consequences of eryptosis can be, parasite clearence, anemia and immunosuppression.

In malaria, eryptotic process has been better studied in infection by *P. falciparum*, which induces oxidative stress in the host red blood cell, promoting the opening of calcium channels and inducing eryptosis. During infection, the parasite, indeed, digests hemoglobin to obtain nutrients and, consequently, hydrogen peroxide is generated, and OH radicals induce oxidative stress (26–28). Therefore, eryptosis in *P. falciparum*-infected red blood cells occurs as a consequence of normal parasite development, with progressive induction of PS exteriorization mediated by increase of cytoplasmic calcium influx in these host cells. It is hypothesized however that the parasite sequesters calcium in its own cytosol, preventing the deflagration of eryptosis during the earlier stages of the intraerythrocytic and allowing the maturation of parasite forms. Parallelly, *P. falciparum* also increases the activity of sphingomyelinase, catalyzing the synthesis of ceramide from sphingomyelin. In turn, increased production of ceramide, even with low concentrations of calcium, can be responsible for eryptotic events, including externalization of phosphatidylserine (17) (**Figure 1**).

Some host pro-apoptotic factors appear to be associated with eryptosis induction during *Plasmodium* infection, such as hematin (29), granzyme B (30), soluble FAS ligand (sFAS-L) (31), anti-erythrocyte (32) and anti-phosphatidylserine antibodies (33–37), as well as some anti-apoptotic factors that are reduced during infection, such as nitric oxide (NO), erythropoietin and vitamin E, and thus can contribute to eryptosis (38–40).

## Pathological and/or Beneficial Roles of Eryptosis in Malaria

Eryptosis also appears to be induced in uninfected red blood cells (**Figure 1**), in infections by *P. yoelii*, *P. berghei* and by *P. falciparum* (41–43). Infected and uninfected red blood cells are likely to suffer eryptosis, the latter case being responsible for a worsening of the anemia caused by the infection. On the other hand, eryptotic red blood cells become refractory to infection, which is a possible mechanism of host defense, thus controlling parasitemia (41, 44). It is believed that eryptosis has an important role in the pathogenesis of malaria, especially in severe anemia, however, this role is still controversial. Severe anemia during malaria can be attributed to the lysis of red blood cells during the blood plasma cycle, or the elimination of uninfected blood cells (45, 46).

In addition, during infection there is an inefficient production of red blood cells by the bone marrow (47, 48), demonstrated by the increasing numbers of reticulocytes in the periphery, consequence of the great loss of red blood cells (49). It is believed that the uptake of red blood cells by macrophages, in addition to directly contributing to their elimination, may contribute to the inefficient production of red blood cells, as it would limit the bioavailability of iron for the erythropoiesis process (50). It has been argued that phosphatidylserine exposure in infected red blood cells may play a role in cytoadherence during infection (51), contributing to the adhesion and sequestration of these cells in the vascular endothelium, as well as for cell aggregation and the formation of a structure containing infected and uninfected red blood cells, called rosette (52–54).

Consequently, the increased exposure of phosphatidylserine in infected red blood cells has harmful effects for patients with malaria: the sequestered and adherent erythrocytes prevent the immune elimination of *Plasmodium* in the spleen; adherence to the endothelium and other cells induces thrombus occlusion, which leads to severe disease; and an a anemia, that is also a main consequence of eryptosis. All events are well characterized in the case of infections by *P. falciparum* and have until now been attributed mainly to exposure on the cell surface of PfEMP1 (*P. falciparum* erythrocyte membrane protein 1). Interestingly, in the case of *P. vivax*, which does not express PfEMP1, there is some evidence that red blood cells infected with *P. vivax* are able to adhere to the endothelial walls, suggesting the participation of phosphatidylserine (55). However, eryptosis in *P. vivax*-infected red blood cells has not yet been studied and it has been demonstrated that, differently from plasma of patients infected with *P. falciparum*, those of *P. vivax* patients are not able to induce eryptosis in uninfected red blood cells, as determined by PS staining (29).

Alternatively, the preference of *P. vivax* to infect reticulocytes, whose PS exposition is observed at the later stages of maturation into red blood cells, could be involved in the cytoadherent phenomena occurring with *P. vivax*-infected red blood cells, contributing to thrombotic events related to malaria, as suggested in sickle cell disease (56). The presence of autoantibodies is frequently reported in patients with malaria, including autoantibodies against phosphatidylserine (33–35, 37). The binding of these antibodies to phosphatidylserine in the outer membrane of eryptotic red blood cells, whether infected or not (34), can promote their lysis due to complement system activation, as well as promote phagocytosis mediated by Fc receptors, present in macrophages (35). The elimination of infected red blood cells contributes to the reduction of parasitemia. However, at the same time that eryptosis eliminates infected cells, it ends up inducing anemia, because through the same mechanisms it promotes the elimination of uninfected red blood cells (35).

Another hypotheses for the contribution of uninfected eryptotic red blood cells is the interaction between phosphatidylserine and the CD36 receptor present in phagocytic cells such as DCs and macrophages, which would recognize eryptotic red blood cells, infected or not, and eliminate them (57). Probably the same can occur with other phosphatidylserine receptors present in phagocytic cells, whose participation in the elimination of apoptotic cells is known, such as TIM-4 (58), MERKT e AXL (59). These data suggest that eryptosis contributes to the severity of anemia during the course of the disease. But eryptosis can be a host mechanism to fight malaria (42). In addition to eliminating infected red blood cells, and consequently interrupting the *Plasmodium* life cycle, eryptosis makes uninfected red blood cells refractory to infection by *P. falciparum*, which preferentially invades non-eryptotic red blood cells, suggesting that the increased eryptosis correlates with a lower parasitic load (42) (**Figure 1**).

Several studies show that the immune response during malaria is deficient (60–63). Some of these works indicate that *Plasmodium* induces a deficient immune response, possibly as a mechanism for the parasite to sustain its life cycle in the human host, allowing the transmission of sexual forms to the mosquito and continuing its life cycle (64). Apoptosis has been described in several human pathologies, infectious or not, related to immunoregulation. Phagocytosis of apoptotic cells would induce a suppressive profile in macrophages and dendritic cells (DCs) (2, 65).

There are no studies showing the direct relationship between eryptosis and immunomodulation during malaria, but Urban and collaborators, in 1999, described that the infected red blood cells that adhere to CD36 on DCs inhibit their maturation. At this time, it was believed that the PfEMP1 protein was responsible for the interaction between the infected red blood cell and DCs, and that in this way the red blood cells would be imitating the immunosuppressive effects induced by apoptotic cells (66). Today we know that eryptotic red blood cells, infected or not, through phosphatidylserine, can interact with CD36 on DCs and macrophages contributing to their elimination, possibly also contributing to the development of a suppressor phenotype in DCs (57) (**Figure 1**).

## Eryptosis in Other Pathologies

Lang and colleagues observed that the red blood cells of patients suffering from thalassemia, sickle cell anemia and glucose-6-phosphate dehydrogenase deficiency are more sensitive to glucose depletion and oxidative stress, and that the red blood cells of patients with sickle cell anemia and glucose-6-phosphate dehydrogenase deficiency are also more sensitive to osmotic shock. Thus, they observed that the red cells of these patients are more likely to undergo eryptotic process (67). The elimination of these infected eryptotic red blood cells occurs by opsonization *via* activation of the complement system, and is one of the mechanisms responsible for the protection observed in these patients against Plasmodium infection (68).

In addition to infections by protozoa of the genus *Plasmodium*, eryptosis has already been described in other infections, such as infection by *Mycoplasma suis* (69) as a representative for hemotrophic mycoplasmas to study anemia pathogenesis caused by pathogens, and during sepsis (70). The same was observed *in vivo* during infection with the *Trypanosoma brucei* (71), and in viral infections such as with LCMV (Lymphocytic Choriomeningitis virus) (72). The production of autoantibodies against phosphatidylserine promotes the elimination of uninfected red blood cells, causing anemia.

In sepsis, the release of molecules derived from pathogens such as peptidoglycans, lipopeptides, α-haemolysin, pyocyanin and listeriolysin are capable of inducing eryptosis, also causing anemia (70). Pyocyanin, a virulence factor of *Pseudomonas aeruginosa*, induces eryptosis through the accumulation of reactive oxygen species in red blood cells (73). The enhanced eryptosis may contribute to an aggravated microcirculatory derangement associated with sepsis (74). An increase in the number of eryptotic red blood cells is also observed in diabetes. This is believed to be due to a by-product of glycolysis, the dicarbonyl compound methylglyoxal (75). Erythrocytes from diabetic patients show increased superoxide dismutase activity and ROS production, factors that favor phospholipid scrambling of the erythrocyte cell membrane (76).

## Concluding Remarks

Eryptosis is an apoptosis-like process that occurs physiologically in senescent red blood cells (15). Infectious processes such as *Plasmodium* infection are capable of inducing eryptosis (21). *Plasmodium* promotes oxidative stress that induces the opening of calcium channels, increasing their concentration in the cytoplasm of red blood cells. Consequently, the increase in calcium induces the exposure of phosphatidylserine in the plasma membrane of red blood cells (26–28).

Thus, eryptosis and particularly phosphatidylserine exposure has two main consequences on the severity of malaria. First, we can discuss eryptosis as a protective mechanism of the host against infection. This is because during *Plasmodium* infection, not only infected red blood cells undergo eryptosis, but also uninfected red blood cells, which become refractory to infection (41–43). We could say that this would be a mechanism of the host to contain the infection. In addition, the presence of antibodies against phosphatidylserine promotes the elimination of infected red blood cells by phagocytes through Fc receptors, which interrupts the parasite cycle, reducing the parasite load (42), similarly to occurring in other infections such as African trypanosomes (71). On the other hand, this same mechanism also promotes cytoadherent phenomena of parasitized red blood cells, as well as the elimination of non-parasitized red blood cells, contributing to the vascular damage and anemia reported in malaria, respectively (35). Moreover, eryptosis may be associated with immunomodulation of the immune response to the parasite, inhibiting the maturation of DCs and promoting a suppressive phenotype in them, that is, inducing an insufficient response to combat the disease (57, 66).

## Author Contributions

AMS and AM conceived the manuscript. AMS, PT and AM wrote the manuscript. AMS, PT and AM participated in the preparation of the manuscript.

## Conflict of Interest

The authors declare that the research was conducted in the absence of any commercial or financial relationships that could be construed as a potential conflict of interest.

## Publisher’s Note

All claims expressed in this article are solely those of the authors and do not necessarily represent those of their affiliated organizations, or those of the publisher, the editors and the reviewers. Any product that may be evaluated in this article, or claim that may be made by its manufacturer, is not guaranteed or endorsed by the publisher.
